# Follicle-stimulating hormone mediates the consumption of serum-derived glycogen by bovine cumulus-oocyte complexes during *in vitro* maturation

**DOI:** 10.14202/vetworld.2021.2512-2517

**Published:** 2021-09-24

**Authors:** Ludymila F. Cantanhêde, Cristiane T. Santos-Silva, Marcelo T. Moura, José C. Ferreira-Silva, Júnior M. B. Oliveira, Daniel N. A. Gonçalves, Álvaro A. C. Teixeira, Valéria Wanderley-Teixeira, Marcos A. L. Oliveira

**Affiliations:** 1 Department of Veterinary Medicine, Federal Rural University of Pernambuco - UFRPE, Recife 52171900, Brazil; 2 Department of Morphology and Animal Physiology, Federal Rural University of Pernambuco - UFRPE, Recife 52171900, Brazil.

**Keywords:** biomolecules, cattle, developmental competence, gonadotropin, granulosa, sera

## Abstract

**Background and Aim:**

Oocyte *in vitro* maturation (IVM) is an appealing approach for several assisted reproductive technologies and dissecting oocyte maturation. Nonetheless, IVM leads to lower developmental competence and usually relies on undefined, serum-containing media. Therefore, biochemical profiling aimed to explore fluctuations in IVM media content during the acquisition of oocyte developmental competence.

**Materials and Methods:**

Bovine cumulus-oocyte complexes (COCs) underwent IVM in TCM199 medium with Earle’s salts, supplemented with 2.0 mM L-glutamine, 10% fetal bovine serum, antibiotics, and 0.05 IU/mL porcine follicle-stimulating hormone (FSH+) or vehicle control (CTL) medium for 22 h.

**Results:**

FSH withdrawal (CTL) diminished several processes associated with the acquisition of oocyte developmental competence, such as reduced cumulus cell expansion, diminished estradiol synthesis (FSH+: 116.0±0.0 pg/mL vs. CTL: 97.6±18.0 pg/mL), and lower oocyte nuclear maturation rate (FSH+: 96.47% vs. CTL: 88.76%). Fresh media formulations (i.e., TCM199 with FSH or vehicle) were indistinguishable under biochemical profiling threshold conditions. Biochemical profiling showed similar total protein and lipid concentrations between groups. Further, total sugar concentrations diminished from fresh media to their post-IVM counterparts, albeit in an FSH-independent manner. Glycogen concentrations remained unaltered after IVM within CTL media, albeit were substantially lower after IVM under FSH+ conditions.

**Conclusion:**

FSH mediates the consumption of serum-derived glycogen by bovine COCs during IVM and implies that serum-free media should contain increased glucose concentrations to facilitate the acquisition of oocyte developmental competence.

## Introduction

Oocytes must undergo nuclear and cytoplasm changes during the acquisition of developmental competence, which is the potential to sustain early embryogenesis compatible with full-term development. Oocyte maturation includes nuclear processes such as germinal vesicle breakdown, nucleolus diffusion, chromatin condensation, and extrusion of the first polar body [1]. The cytoplasmic alterations associated with developmental competence correlate with structural changes and biochemical processes [2-4]. Cytoplasmic changes during the acquisition of developmental competence include protein synthesis [5], protein post-translational modifications [2-4], reorganization of cellular organelles [6], and activation of Ca^2+^-release mechanisms [7].

Both nuclear and cytoplasmic changes during the acquisition of developmental competence *in vivo* rely on the follicular microenvironment, which contemplates the follicular fluid and paracrine factors from multiple neighboring cell types [8,9]. Oogenesis demands several components of the follicular fluid, which includes energy substrates, amino acids, hormones (i.e., steroid and pituitary), cytokines, and growth factors [9-12]. The follicle-stimulating hormone (FSH) is one of the better understood factors to promote oocyte developmental competence [8,13,14]. For instance, estradiol is a steroid hormone found in the follicular fluid, and its concentration increases during follicle development [15,16]. This estradiol increase spawns from FSH-mediated cumulus cell signaling [14]. In sum, oocytes attain developmental competence by a plethora of interactions among cumulus cells, the oocyte, and the follicle environment [8,9,17].

Oocyte *in vitro* maturation (IVM) recapitulates, at least partially, cellular and molecular processes that occur during oocyte maturation *in vivo* [17-19]. Despite efficient nuclear maturation under most experimental IVM conditions [20,21], oocytes subject to IVM display lower developmental competence than those matured *in vivo* [17]. Oocytes under IVM fail to acquire full cytoplasmic maturation, and IVM media must upgrade to improve developmental competence. Synthetic IVM media allowed successful livestock oocyte IVM and *in vitro* embryo production (IVP) [17]. Basal media (e.g., TCM199) used for IVM and IVP have formulations optimized for somatic cell culture [22]. Therefore, these basal media require substantial supplementation with known factors to promote oocyte maturation and the acquisition of developmental competence during IVM. FSH is one of the essential components of IVM media [8,14]. Numerous reports have functionally demonstrated the importance of FSH for the acquisition of oocyte developmental competence [14,23-26]. Since FSH regulates (directly and indirectly) several metabolic processes in oocytes and cumulus cells during IVM [8,14], FSH withdrawal may reveal insights into cumulus-oocyte complexes (COCs) metabolic activities during IVM. This approach would allow distinguishing between processes required for developmental competence (using IVM medium supplemented with FSH) from those happening under basal needs for cellular physiology (under FSH-free IVM medium).

Therefore, the study aimed to perform biochemical profiling under FSH-containing and FSH-free IVM conditions and prospecting modulations in IVM media associated with the acquisition of oocyte competence in cattle.

## Materials and Methods

### Ethical approval

The experiment was approved by the Ethics Committee (Comissão de Ética e Experimentação Animal – CEUA) from the Universidade Federal Rural de Pernambuco (Protocol 060/2013).

### Oocyte maturation

The IVM of bovine COCs was performed as previously described by Moura *et al*. [27]. Briefly, ovary collection was at local abattoirs (Pernambuco state, Brazil) and transported in saline solution (0.9 % NaCl) containing 10 IU/mL penicillin and 10 μg/mL streptomycin (Gibco, Waltham, MA, USA) at 35°C within 3 h after slaughter. The COCs were retrieved from 2 to 8 mm follicles using 10 mL syringes coupled with 19 G needles into H-IVM medium (TCM199 medium with Hank’s salts [Sigma-Aldrich, St. Louis, MO, USA] supplemented with 2 mM L-glutamine [Sigma-Aldrich, St. Louis, MO, USA], 10% fetal bovine serum [FBS] [Gibco, Waltham, MA, USA], and 0.05 μg/mL gentamicin sulfate [Gibco, Waltham, MA, USA]).

Retrieved COCs with homogeneously granulated oocyte cytoplasm and at least three complete cumulus cell layers were used for IVM [27]. The COCs were randomly distributed into FSH-containing IVM medium (FSH+; 0.05 IU/mL pFSH – Sigma-Aldrich, St. Louis, MO, USA) or the vehicle-containing IVM medium (control – CTL). The IVM medium was formulated with TCM199 with Earle’s salts supplemented with 2 mM L-glutamine, 10% FBS, 10 IU/mL penicillin, and 10 mg/mL streptomycin (Gibco, Waltham, MA, USA). All IVM media were adjusted for pH (7.2-7.4) and osmolarity (260-280 mOsm). Experimental groups were formed by pools of 20-25 COCs [28] per 150 μL of IVM medium and further incubated with 5% CO_2_, saturated humidity at 38.5°C for 22 h. The media after IVM were transferred to microtubes and centrifuged at 2,000 g for 5 min. Moreover, the supernatant was collected and stored at −20°C, alongside with fresh media (i.e., not used for IVM) for further estradiol concentration analysis profiling.

### Analysis of oocyte nuclear maturation

The COCs were denuded after 22 h of IVM. All COCs were initially washed and kept in 0.2% hyaluronidase (Sigma-Aldrich, St. Louis, MO, USA), then further incubated in hyaluronidase solution and gentle pipetting for 5 min. Oocytes were washed from cumulus cells in H-IVM media. The nuclear maturation efficiency was determined by the number of oocytes with visible polar bodies.

### Estradiol analysis

The estradiol concentration was determined by the immunoassay Access 2 (Beckman Coulter, Brea, CA, USA) at the CENAPESQ Center, UFRPE. The analysis was carried out with the Estradiol 2 X 50 DET – BC kit and the Estradiol CAL S0-S5 Access.

### Biochemical profiling

Biochemical profiling was carried out using a Bel photonics SP 2000 UV spectrophotometer (ABM Italy, Milan, Italy). The total protein analysis was performed with the Bradford assay with the Coomassie blue [29]. A 100 μL volume of each sample was mixed with 5 mL Bradford solution for 2 min and determined in the spectrophotometer with a 595 nm reads.

The total glycogen, lipid, and sugars concentrations were determined by the method described by Santos Silva *et al*. [29]. A volume of 200 μL sodium sulfate and 800 μL chloroform-methanol (1:1) were mixed with 200 μL of each IVM media sample. After homogenizing, samples were centrifuged at 2000 g for 2 min. The precipitate was used for glycogen analysis, while the supernatant was transferred to another microtube for the assessment of sugars and lipids. The total lipid content was determined by spectrophotometry using phosphoric acid and vanillin [29], while total sugar content and glycogen concentration were analyzed using sulfuric acid and anthrone [29]. Absorbance was read at 625 nm, and five replicates were used in duplicate for each IVM media (FSH supplemented – fresh and after IVM; vehicle control – fresh and after IVM).

### Statistical analysis

The estradiol data (pg/mL) were initially subject to logarithmic transformation of the base 10, while the glycogen and sugars data were subject to radical and logarithmic transformations, respectively. All continuous data were described as means, standard deviation, and range. The assessment of the data for normality was determined by the Shapiro–Wilk test. The data were subject to the F-test or ANOVA, while the mean comparisons were carried out using Tukey’s HSD test or the Student-Newman-Keuls test [30]. The efficiency of oocyte nuclear maturation rates was analyzed by the Chi-square test. The IBM SPSS Statistics software version 23.0 (IBM, Armonk, NY, USA) was used for the statistical analysis. The significance level was 5%.

## Results

**Figure-1:**
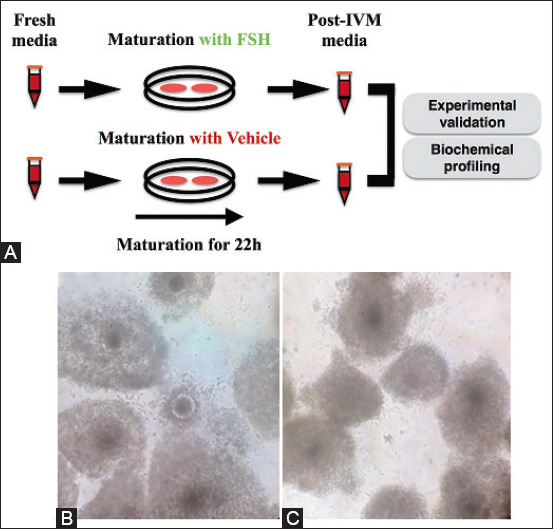
Experimental design and cumulus cell expansion analysis under varying follicle-stimulating hormone (FSH) conditions. (A) *In vitro* maturation (IVM) media collection before and after oocyte IVM for 22 h. Oocytes harvested for experimental design validation and IVM media were subject to both estradiol quantification (experimental design validation) and composition analysis (biochemical profiling). Expansion of cumulus cells of cumulus-oocyte complexes after 22 h of IVM under FSH containing – FSH+ (B) and control vehicle-containing IVM media (C).

To demonstrate the potential of the experimental design, cumulus cell expansion, estradiol synthesis, and oocyte nuclear maturation were recorded after IVM of bovine COCs with or without FSH supplementation (Figure-1). The cumulus cell expansion was substantially more intense in the FSH+ group than in the non-treated CTL (Figure-1). Moreover, FSH deprivation diminished nuclear maturation efficiency determined by polar body extrusion (Table-1). Estradiol concentration measurement demonstrated FSH-mediated modulation of cumulus cell physiology (Table-2). The presence of FSH in IVM media formulation did not affect estradiol levels in fresh media (i.e., FSH+ and CTL). Further, IVM media from the CTL group showed similar concentrations between the fresh and post-IVM samples. In turn, estradiol level increased in FSH+ media after IVM in comparison to its fresh counterpart sample (Table-2).

**Table-1:** Nuclear maturation rates after exposure of bovine cumulus-oocyte complexes to FSH supplementation (FSH+) or its vehicle (CTL) during *in vitro* maturation for 22 h.

Group	Cumulus-oocyte complexes	Oocytes with polar body	Maturation rate (%)
FSH	170	164	96.47^A^
CTL	178	158	88.76^B^

Five replicates. Follicle-stimulating hormone

FSH=FSH-containing IVM media.

CTL(control)=Vehicle-containing IVM media

Different superscript letters (A, B) denote statistical difference by the Chi-square test (p<0.05)

**Table-2:** Estradiol concentration (pg mL^-1^) before and after exposure of bovine cumulus-oocyte complexes to FSH or its vehicle (control) during *in vitro* maturation for 22 h.

Statistical parameter	FSH		Control	
	
	Before *in vitro* maturation	After *in vitro* maturation	Before *in vitro* maturation	After *in vitro* maturation
Mean±SD	58.3±8.6^B^	116.0±50.8^A^	56.0±0.0^B^	97.6±18.0^B^
Range	49.0-66.0	79.0-174.0	56.0-56.0	79.0-115.0

SD=Standard deviation; different superscript letters (^A, B^) denote statistical difference (p<0.05). F-test, Tukey’s HSD test, and Student-Newman-Keuls test. FSH=Follicle-stimulating hormone

Biochemical profiling in maturation media (before and after IVM in both FSH+ and CTL groups) prospected modulations in composition associated with the acquisition of oocyte developmental competence (Table-3). The screening on IVM media samples analyzed total protein, lipids, sugars, and glycogen concentrations. IVM media formulation (FSH+ or CTL) did not lead to any variation in biochemical parameters (i.e., below detection threshold). Both total protein and lipid contents were similar between samples, irrespectively of FSH supplementation or IVM. Sugar content diminished after IVM, albeit in an FSH-independent manner. Fluctuations in glycogen concentration between fresh CTL media and its post-IVM sample did not reach the statistical threshold. Nonetheless, the glycogen concentration lowered after IVM under FSH+ conditions, thus suggesting FSH-mediated consumption (Table-3).

**Table-3:** Media composition before and after exposure of bovine cumulus-oocyte complexes to FSH or its vehicle (control) during *in vitro* maturation for 22 h.

Content	FSH		Control	
	
	Before *in vitro* maturation	After *in vitro* maturation	Before *in vitro* maturation	After *in vitro* maturation
Protein	22.0±1.4^A^	22.9±2.6^A^	21.5±4.9^A^	22.2±2.9^A^
Lipids	26.1±0.7^A^	27.0±2.2^A^	26.2±3.4^A^	25.9±2.2^A^
Sugars	146.8±35.9^A^	43.4±25.9^B^	146.1±32.6^A^	41.3±19.1^B^
Glycogen	14.8±6.0^A^	2.2±4.1^B^	12.1±4.9^A^	8.9±5.5^A^

SD=Standard deviation; different superscript letters (^A, B^) denote statistical difference (p<0.05). F-test, Tukey’s HSD test, and Student-Newman-Keuls test

## Discussion

Repeated collection of immature oocytes from donor cows largely increases the number of transferable IVP embryos per cow in any given stretch of time [17]. However, this increased number of embryos per cow is counterbalanced by the diminished developmental competence of IVM-derived oocytes (and resulting IVP embryos) in comparison to oocytes matured *in vivo* [17,31]. Therefore, IVM conditions must improve to mimic *in vivo* conditions and enhance oocyte developmental competence.

In this work, the effect of FSH on the expansion of cumulus cells, estradiol production, and oocyte nuclear maturation was observed *ex vivo*, thus reinforcing the seminal effects of this hormone on cellular hallmarks of oocyte maturation [32-34]. Cumulus cells mediate FSH signaling pathways that contribute to the acquisition of developmental competence [14,35-37]. The gene expression profile and physiology of cumulus cells are subject to extracellular cues and interactions with the oocyte [38,39]. When supplemented in IVM media, FSH promotes the increase of cumulus cell expansion [40], resumption of meiosis, and steroid hormone production [41-43].

The experimental approach envisioned that removal of a critical component of the IVM media (i.e., FSH) would render oocytes non-competent and impair biochemical processes required for developmental competence. Further, such condition would reveal processes associated with developmental competence by the analysis of medium composition before and after IVM. The results at the cellular level validated the experimental design and unequivocally allowed to prospect for media compositions fluctuations associated with the acquisition of oocyte developmental competence. Biochemical profiling revealed the dynamic nature of IVM media composition during oocyte IVM.

Total protein and lipid concentrations remained constant among all experimental conditions. Several proteins play pivotal roles during oocyte maturation, such as the proteins that form the maturation promoting factor complex, and signaling pathways such as mitogenic activated protein kinase, epidermal growth factor related, and protein kinase A [5,44-46], respectively. Lipids may be toxic or beneficial during IVM [43,47-49]. The inability to identify differences in total protein and lipid levels may be due to the detection thresholds or a balance between catabolism and anabolism. Additional analyses focusing on candidate proteins/lipids or unbiased genome-wide tools of greater resolution may reveal further details of IVM media modulations.

Sugar levels lowered after IVM in an FSH-independent manner, thus suggesting that IVM actively demands energy substrates, most likely by cumulus cells, due to their abundance and energy substrate preferences. The metabolic demands of bovine COCs also fluctuate during IVM, as demonstrated by measurements of glucose and pyruvate uptake [50]. Cumulus cells rely on different energy sources (glucose, lactate, and pyruvate) for energy metabolism than oocytes (lactate and pyruvate) [51]. In sum, energy supply is paramount for cellular activity during IVM and the acquisition of oocyte developmental competence [10,52,53].

The most notable observation in this study was glycogen consumption under the influence of FSH. Since the basal medium TCM199 does not contain animal-derived components, FBS was the source of glycogen in IVM media. The IVM of COCs under CTL medium conditions did not affect glycogen concentrations, thus ruling out degradation during media incubation *in vitro* or metabolism by cumulus cells (FSH-independent metabolism). More importantly, this increased energy demand was restricted to the acquisition of oocyte developmental competence. Several non-mammalian model organisms accumulate intracellular glycogen or glycogen phosphatase during oocyte maturation [53,54]. Notwithstanding, mural granulosa and cumulus cells of macaque females have the detectable expression of the glycogen phosphatase enzyme [55,56], thus suggesting the potential to convert glycogen into glucose-6-phosphate, which can be later metabolized by glucose metabolism pathways. However, further research should shed light on glycogen concentrations in IVM media, glycogen metabolism by COCs, and its potential connection to the acquisition of oocyte developmental competence.

## Conclusion

FSH mediates the consumption of serum-derived glycogen by bovine COCs during oocyte IVM and implies that serum-free media should contemplate increased energy substrates to compensate for glycogen as an energetic metabolism substrate.

## Authors’ Contributions

LFC: Study design, data collection, data interpretation, and manuscript preparation. CTS: Data collection and interpretation. MTM: Study design, data collection, data interpretation, manuscript preparation, and funding support. JCF: Data collection and interpretation. JMBO: Data interpretation and statistical analysis. DNAG: Data collection and interpretation. AACT: Study design, data interpretation, and project supervision. VW: Study design, data interpretation, and project supervision. MALO: Study design, data interpretation, manuscript preparation, funding support, and project supervision. All authors have read and approved the final manuscript.
